# Effect of pentoxifylline on endothelial dysfunction, oxidative stress and inflammatory markers in STEMI patients

**DOI:** 10.2144/fsoa-2023-0266

**Published:** 2024-05-15

**Authors:** Asmaa Saeed, Mohamed Moustafa Farouk, Nagwa Ali Sabri, Mohamed Ayman Saleh, Marwa Adel Ahmed

**Affiliations:** 1 Department of Clinical Pharmacy, Faculty of Pharmacy, Ain Shams University, Cairo, 11566, Egypt; 2 Department of Cardiology, Faculty of Medicine, Ain Shams University, Cairo, 11591 Egypt

**Keywords:** acute coronary syndrome, endothelial dysfunction, inflammation, malondialdehyde, oxidative stress, pentoxifylline, soluble vascular cell adhesion molecule 1, ST-elevation myocardial infarction

## Abstract

**Aim:** ST-elevation myocardial infarction (STEMI) patients suffer higher mortality and adverse outcomes linked to endothelial dysfunction (ED). **Methods:** 43 patients were randomized to pentoxifylline (PTX) 400 mg thrice daily (n = 22) or placebo (n = 21). Soluble vascular cell adhesion molecule-1, malondialdehyde, interleukin-1 (IL-1), interleukin-6 (IL-6), high-sensitivity C-reactive protein (hs-CRP) and tumor necrosis factor-α (TNF-α) were assessed at baseline and 2 months. **Results:** After 2 months, no significant difference was observed in markers' levels between the 2 groups. However, a within-group comparison revealed a statistically significant change in hs-CRP in the PTX group (10.057 (9.779–10.331) versus 9.721 (6.102–10.191)), p = 0.032. **Conclusion:** PTX for 2 months in STEMI patients was safe and well-tolerated but had no significant detectable effect on ED, oxidative stress or inflammatory markers.

**Clinical Trial Registration:** NCT04367935 (ClinicalTrials.gov)

## Plain language summary

This study examined the effect and the safety of a drug called pentoxifylline in patients who have recently had a heart attack. Pentoxifylline can possibly reduce inflammation and is used for patients with blood flow issues. The study involved 43 participants, 22 receiving pentoxifylline and 21 receiving a placebo for 2 months. We measured different markers related to inflammation and heart health before and after. Overall, there was no significant difference between the groups, but patients who received pentoxifylline experienced less inflammation according to only one of the markers measured. This study concluded that the prescription of pentoxifylline after a heart attack is safe, well-tolerated and without notable side effects. Still, we recommend larger and longer studies to be sure of its effect.

## Abstract (plain language summary)

Despite the advances in treatment modalities, research is still ongoing for therapies to improve outcomes in STEMI patients.

Pentoxifylline (PTX), a drug with anti-inflammatory and antioxidant potential, was evaluated in this study for its effect on ED, oxidative stress, and inflammation biomarkers in STEMI patients.

The use of PTX in our study had a neutral effect on ED, oxidative stress, and inflammation but with a significant decrease in the level of hs-CRP in the PTX group.

Our study provided a basis for the safe and tolerable use of PTX in STEMI patients thus providing the opportunity to safely investigate the use of PTX in different cardiac settings.

Future studies on a larger scale are warranted to fully establish the effect of PTX on this patient population.

Ischemic heart diseases (IHDs) remain the main culprit for worldwide mortality, being responsible for 16% of global deaths in 2019 [1]. Unstable heart ischemia known as acute coronary syndrome (ACS) is associated with a high risk of morbidity and mortality despite the advancement in revascularization and secondary prevention strategies [2]. Up to 52% of ST-segment elevation myocardial infarction (STEMI) patients suffer from major adverse cardiac events (MACEs) such as recurrent anginal pain, reinfarction, rehospitalization, stroke, unplanned revascularization and mortality and around 10% die within 1 year of the event [3,4]. The demand for research into new treatment strategies for STEMI continues since the risk of recurrence, heart failure development and long-term mortality post-myocardial infarction (MI) remains high [5].

The endothelial dysfunction (ED), oxidative stress and inflammation are all interlinked concepts that have been connected to cardiovascular morbidity and mortality [6]. Endothelial dysfunction in ACS has been linked to larger infarcted areas, worse left ventricular ejection fractions (LVEF), future MACEs and cardiac deaths [7,8]. Interestingly, normalization of the ED within 8 weeks after the ACS event was linked to fewer future cardiovascular events [9].

Oxidative stress contributes not only to cardiovascular events but also to the development of ED [10]. STEMI patients treated with percutaneous coronary intervention (PCI) who had a major cardiac event later on, had high levels of oxidative stress markers [11].

Additionally, the activated immune system post-MI contributes to positive repair mechanisms of the heart muscle but sustained activation and inflammation result in the negative ventricular remodeling that can lead to heart failure [12,13]. Continued inflammation after an ACS event was also linked to higher rates of MACEs and mortality [14,15].

Pentoxifylline (PTX) is a methylxanthine derivative that enhances red blood cells deformability, decreases platelet aggregation and lowers blood viscosity, thus it improves the microcirculation and oxygenation of ischemic tissue. It has been shown to inhibit several inflammatory cytokines secretion such as interleukin-1 (IL-1), interleukin-6 (IL-6) and tumor necrosis factor-α (TNF-α) consequently inhibiting adhesion between the leukocytes and endothelium while also reducing free radical formation [16,17].

The beneficial effects of PTX on endothelial function, oxidative stress and inflammation have been elucidated in different populations and settings [17]. Pentoxifylline is generally safe with few side effects, most commonly gastrointestinal symptoms such as nausea [18]. Reports of bleeding and/or increased prothrombin time have been noted with PTX use [19]. However, PTX was found to have no additional platelet inhibitory effect in diabetic patients who were receiving dual-antiplatelet therapy with aspirin plus clopidogrel [20]. In addition, the administration of PTX in NSTEMI patients in two different trials was well tolerated, with only a few patients in one trial complaining of nausea and headache that did not require a discontinuation of treatment [21,22].

Our research question was whether PTX would elicit a significant improvement in the ED, oxidative stress and inflammatory status in STEMI patients. To the best of our knowledge, this is the first trial to assess the use of PTX in STEMI patients regarding its effect on endothelial function, oxidative stress, inflammation and MACEs development all together.

## Materials & methods

### Study design

This study was a randomized, placebo-controlled, parallel, single-blinded study. The clinical trial reporting followed the CONSORT 2010 guidelines [23].

### Setting

Recruitment of patients took place at the Coronary Care Unit (CCU) at Ain Shams University Hospitals, Cairo, Egypt from 24th January 2021 to 25th January 2022. The study was concluded with the last patient finishing the required follow-up period on 25th March 2022.

### Ethics

The study protocol was revised and approved by the Research Ethics Committee for Experimental and Clinical Studies at Faculty of Pharmacy, Ain Shams University (ENREC – ASU no. 289) and was performed in accordance with the declaration of Helsinki and the International Conference on Harmonization Guidelines for Good Clinical Practice [24,25]. A written informed consent was obtained from all patients prior to participation without any obligation in case of withdrawal. The study was registered with clinicaltrials.gov registration number: NCT04367935.

### Patients

Patients with MI were screened for eligibility under the following inclusion and exclusion criteria:

#### Inclusion criteria

Patients aged 18–80 years old presenting with STEMI diagnosis who approved to participate and gave an informed consent. The diagnostic criteria of the European Society of Cardiology (ESC) were followed in which symptoms pertaining to myocardial ischemia plus ECG changes showing ST-segment elevation is recorded. ST-segment elevation should be persistent for >20 min and evident in at least two contiguous leads, at ≥2.5 mm in men <40 years, ≥2 mm in men ≥40 years, or ≥ 1.5 mm in women in leads V_2_–V_3_ and/or ≥1 mm in the other leads in the absence of left ventricular hypertrophy or left bundle branch block [26].

#### Exclusion criteria

Known allergy to PTX or any methylxanthine derivate, patients using PTX before the trial, heart failure NYHA class III or IV, serum creatinine level ≥2 mg/dl or known kidney disease, active bleeding, platelet count <100 × 10^3^/μl, PRECISE-DAPT (PREdicting bleeding Complications In patients undergoing Stent implantation and subsEquent Dual Antiplatelet Therapy) score ≥25, major surgery or trauma within 1 month, recent cerebral and/or retinal hemorrhage within 3 months, known liver disease, patients receiving any medication known to interact with PTX, patients on any type of anticoagulant and patients with recent ACS event in the past 6 months. Patients with severe left ventricular dysfunction (LVEF <30%) were also excluded to avoid the confounding effect of the potentially high levels of sVCAM-1 and inflammatory markers in those patients to our study [27,28].

Overall, 83 patients were assessed for eligibility. Only 50 patients were eligible out of which 43 patients completed the study.

### Treatment

All included patients have been treated with primary PCI with routine medications including aspirin loading dose of 300 mg then maintenance of 75 mg/day, ticagrelor loading dose of 180 mg/day then maintenance of 90 mg twice per day or clopidogrel loading dose of 600 mg then maintenance of 75 mg/day, enoxaparin 0.5 mg/kg bolus IV then 1 mg/kg subcutaneous every 12 h for the duration of hospital stay, tirofiban was used in cases of large thrombus or evidence of no-reflow in dose of 25 μg/kg IV over 3 min. then maintenance infusion of 0.15 μg/kg/min for no more than 18 h. Therapy with beta blockers, statins and angiotensin-converting enzyme inhibitors (ACEIs) was initiated in all patients unless there were contraindications. An aldosterone antagonist, trimetazidine or ivabradine were prescribed to select patients according to their clinical condition per the guidelines of the ESC [29].

### Randomization

Eligible patients were randomly assigned to one of two groups in a 1:1 ratio. Group I (n = 22); received 400 mg sustained release (SR) tablets of PTX three times daily in addition to standard treatment for 2 months. Group II (n = 21); received standard treatment in addition to matching placebo three times daily for 2 months. For both groups, the PTX tablets or placebo tablets were always started within 24 h of the event and before patient discharge. The random allocation sequence was generated using the random allocation software with a block randomization of block size 4 with no stratification.

Pentoxifylline was administered as Trental^®^ 400 mg SR tablets, manufactured by Sanofi pharmaceutical company, Paris, France. Matching placebo tablets in color and size were manufactured by Nawah Scientific Research Center, Cairo, Egypt. The oral route of administration was more convenient as opposed to intravenous route since the drug was to be continued on an outpatient basis.

### Clinical laboratory measurements

Baseline data for all eligible patients were collected, including a detailed medical history, demographic data and disease-related information such as type of intervention, result of PCI, history of previous events, baseline PRECISE-DAPT score, LVEF% and standard laboratory parameters such as lipid profile, serum creatinine, aspartate transaminase (AST), alanine transaminase (ALT), complete blood count (CBC), random blood glucose, glycated hemoglobin (HbA1c), creatinine clearance, creatine kinase and international normalized ratio (INR).

Five milliliters of blood were collected from the patients at baseline and at the end of the 2-month follow-up period for evaluation of the required laboratory parameters. The blood was collected in gel tubes with no additives, centrifuged and the serum separated was stored in Eppendorf tubes in a -80 °C refrigerator for evaluation of the outcome markers. The study outcome markers (sVCAM-1, MDA, IL-1, IL-6, hs-CRP and TNF-α) were measured with enzyme-linked immunosorbent assay (ELISA) technique using commercially available kits and the analysis was performed by a specialized laboratory technician who was blinded to the groups.

### Follow-up

Patients were followed up by the principal investigator and the treating physician every 2 weeks for 2 months to monitor tolerability i.e., occurrence of PTX side effects. Also, monitoring of efficacy through recording of MACEs including rate of re-hospitalization, second event, death, stroke, recurrent anginal pain or unplanned revascularization [3]. Evaluation of compliance with the intervention drugs was performed by pill count. Counselling of the patients regarding their medication use and appropriate diet was given during follow-up, as well as counselling regarding any drug-related problems encountered.

### Study outcomes

Between-group comparison of the reduction of sVCAM-1 level from baseline until 2 months as a marker of ED was the primary outcome of the trial. The secondary outcomes were between-group comparison of the reduction of MDA, as a marker for oxidative stress, between-group comparison of the reduction of the inflammatory markers hs-CRP, TNF-α, IL-1 and IL-6 and evaluation of intervention drug's efficacy and tolerability through monitoring of patients' development of MACEs and side effects during drug administration, respectively.

### Statistical analysis

Sample size was calculated using PASS program, setting the type-1 error (α) at 0.05. Results from previous study were used as a reference, where the administration of PTX (400 mg three times per day) for 2 months to patients with Coronary Artery Disease (CAD) resulted in a significant change (p < 0.05) in the level of sVCAM-1 in the treatment group compared with the control group [30]. It was observed that the mean change in sVCAM-1 in treatment group was -298.27 ± 39.061 versus -100.29 ± 10.82 in the control group. Based on this, a sample size of 20 cases per group (40 total) will achieve 100% power to detect the observed difference.

Statistical analysis was performed using SPSS (Statistical Package for Social Sciences Inc., IL, USA) version 23.

Continuous data were described using means and standard deviations if normally distributed or median and interquartile ranges if not normally distributed. The Shapiro–Wilk test was used as a test for normality with most of the data turning out to be non-normally distributed. Levene's test was used to test the equality of variances when needed. Categorical data were described using numbers and percentages and analyzed using either Chi-square or Fischer's exact test according to the fulfilment of the test's assumptions.

For the between-group comparison, either unpaired *t*-test or Mann–Whitney test was used according to the normality. For the within-group comparison, Wilcoxon-signed rank test was the most appropriate to use. Bonferroni correction was then applied by multiplying the resulting p-values of the group comparisons by four to reduce the chances of a false positive results due to multiple comparisons.

The two-sided p-value was used with a value <0.05 considered significant.

## Results

**Figure 1.**
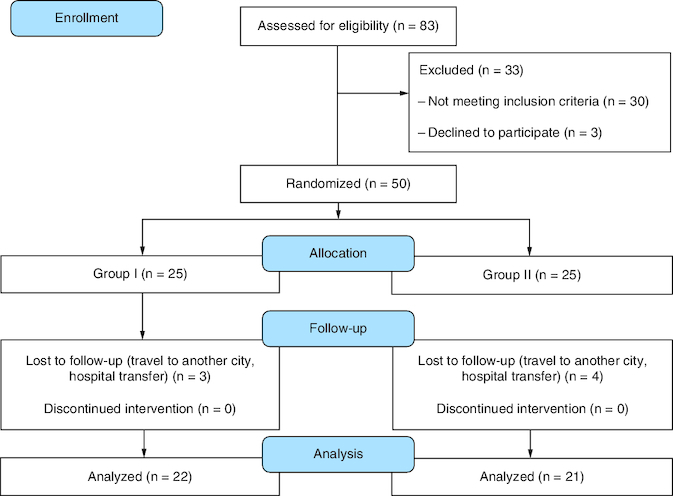
Study flow diagram.

Out of the 83 patients assessed, only 50 were eligible for participation and underwent randomization. After seven patients were lost to follow-up, the study continued with 43 patients (22 patients in the drug group and 21 patients in the placebo group). The study flow diagram is shown in Figure 1.

### Demographic data & baseline clinical characteristics

No statistically significant difference was found between the two groups regarding the baseline characteristics including demographics, clinical characteristics, laboratory data, medical history and disease-related information as shown in Table 1.

**Table 1.** Comparison of the baseline characteristics for the drug and placebo groups.

Characteristics	Drug group (n = 22)	Placebo group (n = 21)	p-value
Demographics Age (years) Gender, male BMI (kg/m^2^)	57.5 (49–60.5) 18 (81.8%) 29.079 ± 5.023	55 (46.5–64.5) 17 (81%) 28.226 ± 3.675	0.77^‡^ 1.00^§^ 0.53^¶^
Smoking status, smoker	10 (45.5%)	11 (52.4%)	0.65^#^
Pack-year history	0.00 (0.00–27.00)	6 (0.00–35)	0.65^‡^
10-year ASCVD risk	15 (5.975–21.8)	12 (5.55–19.9)	0.87^‡^
Previous events, n 0 1 2	18 (81.8%) 4 (18.2%) 0 (0%)	19 (90.5%) 0 (0%) 2 (9.5%)	0.053^§^
Multi- vs single-vessel disease Multi-vessel disease Single-vessel disease	9 (40.9%) 13 (59.1%)	3 (14.3%) 18 (85.7%)	0.052^#^
Location of MI Anterior MI Non-anterior MI	16 (72.7%) 6 (27.3%)	14 (66.7%) 7 (33.3%)	0.67^#^
LVEF%	41.5% (37.5–47.75%)	40% (35–46. 5%)	0.33^‡^
Killip class 1 ≥2	20 (90.9%) 2 (9.1%)	20 (95.2%) 1 (4.8%)	1.00^§^
PRECISE-DAPT score Very low Low Moderate	13 (59.1%) 6 (27.3%) 3 (13.6%)	8 (38.1%) 7 (33.3%) 6 (28.6%)	0.33^§^
Laboratory data Serum creatinine Creatinine clearance ALT AST Total leukocytic count Hemoglobin Platelet count Total cholesterol HDL LDL Triglycerides Random blood glucose INR Total creatine kinase Creatine kinase – MB HbA1c	0.8 (0.8–1.025) 112.545 ± 34.266 38 (23–45) 66 (27.25–183.75) 10.25 (8.15–13.15) 14.223 ± 1.899 245 (217.75–307.25) 194.91 ± 40.734 38.5 (31.5–44.5) 126.5 ± 35.427 141.5 (95.75–194.75) 172.5 (136.5–226.5) 1.055 (1–1.103) 437 (170.5–1555.75) 58.514 (21.75–179.5) 5.8 (4.925–6.671)	1 (0.85–1.1) 97.196 ± 28.518 42 (22–71) 82 (38–181.5) 9.3 (6.6–14.25) 14.152 ± 1.690 268 (229.5–306) 204.048 ± 47.482 39 (33.4–45) 137.705 ± 45.828 115 (99–141.5) 141 (108.5–216.5) 1.1 (1–1.22) 837 (361–1743.34) 72 (42.5–216.5) 5.9 (5.35–8.8)	0.10^‡^ 0.10^¶^ 0.19^‡^ 0.30^‡^ 0.42^‡^ 0.90^¶^ 0.70^‡^ 0.50^¶^ 0.72^‡^ 0.35^¶^ 0.18^‡^ 0.26^‡^ 0.36^‡^ 0.17^††^ 0.36^††^ 0.46^††^
Medical history Hypertension Diabetes mellitus Stroke	9 (40.9%) 13 (59.1%) 1 (4.5%)	12 (57.1%) 9 (42.9%) 0 (0%)	0.22^#^ 0.22^#^ 0.51^§^
Stent type BMS DES	2 (9.1%) 20 (90.9%)	1 (4.8%) 20 (95.2%)	1.00^§^
Stents per patient, n 0 1 2	0 (0%) 17 (77.3%) 5 (22.7%)	1 (4.8%) 15 (71.4%) 5 (23.8%)	0.86^§^
Medication history before admission Aspirin Plavix Ticagrelor Beta-blockers ACEI CCB Statins Insulin OHG	3 (13.6%) 2 (9.1%) 1 (4.5%) 2 (9.1%) 5 (22.7%) 2 (9.1%) 2 (9.1%) 3 (13.6%) 8 (36.4%)	4 (19%) 4 (19%) 0 (0%) 5 (23.8%) 4 (19%) 0 (0%) 3 (14.3%) 4 (19%) 7 (33.3%)	0.70^§^ 0.41^§^ 1.00^§^ 0.24^§^ 1.00^§^ 0.49^§^ 0.66^§^ 0.70^§^ 0.84^#^
In-hospital and discharge medications Antiplatelets Clopidogrel, n (%) Ticagrelor, n (%) Trimetazidine Ivabradine Aldosterone antagonist	14 (63.6%) 8 (36.4%) 4 (18.2%) 3 (13.6%) 12 (54.5%)	19 (90.5%) 2 (9.5%) 5 (23.8%) 3 (14.3%) 7 (33.3%)	0.037^†^^,^^#^ 0.72^§^ 1.00^§^ 0.16^#^

Data are expressed as mean ± SD, number (%), or median (interquartile range).

† A p-value 0.05 denotes significant difference.

‡ Mann–Whitney *U* test.

§ Fisher's exact test.

¶ Unpaired *t*-test.

# Pearson Chi-square test.

†† Multiple Imputation then Mann–Whitney *U* test.

ACEI: Angiotensin-converting enzyme inhibitor; ALT: Alanine transaminase; AST: Aspartate transaminase; ASCVD: Atherosclerotic cardiovascular disease; CCB: Calcium channel blocker; HbA1c: Glycated hemoglobin; HDL: High density lipoprotein; INR: International normalized ratio; IQR: Interquartile range; LDL: Low density lipoprotein; LVEF: Left ventricular ejection fraction; MI: Myocardial infarction; n: Number; OHG: Oral hypoglycemic; PRECISE-DAPT: PREdicting bleeding Complications In patients undergoing stent implantation and subsequent dual antiplatelet therapy; SD: Standard deviation.

A significant positive correlation was found between the baseline levels of sVCAM-1 in the present study and the age of the participants (r = 0.446, p = 0.003).

### Effect of pentoxifylline on the study's biomarkers

**Figure 2.**
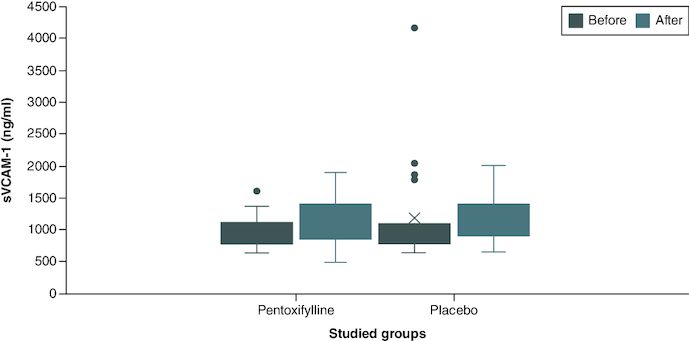
Comparison of sVCAM-1 level between group I and group II at baseline and at the end of the study. Group I (n = 22): patients received 400 mg tablet of PTX thrice daily in addition to their standard treatment for 2 months. Group II (n = 21): patients received placebo tablets in addition to their standard treatment for 2 months. The p-value of end of study level of sVCAM-1 in group I versus group II = 1.00. n: Number; PTX: Pentoxifylline; sVCAM-1: Soluble vascular cell adhesion molecule-1.

The baseline serum concentration of all biomarkers (sVCAM-1, MDA, IL-1, IL-6, hs-CRP and TNF-α) were non-significantly different among the two study groups. At the end of the study, no statistically significant difference could still be observed regarding all the above biomarkers when the serum concentration was compared between the two groups. However, comparison of the within-group change demonstrated a statistically significant decrease in the hs-CRP of the drug group only 10.057 (9.779–10.331) versus 9.721 (6.102–10.191), p-value = 0.032, with no statistically significant difference observed with the other biomarkers as shown in Table 2 & Figure 2.

**Table 2.** Comparison of serum concentration of the study biomarkers for both drug and placebo groups.

Parameter	Drug group (n = 22)	Placebo group (n = 21)	p-value
sVCAM-1 (ng/ml) Baseline End point p-value of within group	967.542 (783.083–1104.225) 1203.889 (850.614–1411.699) 0.052^§^	922.787 (783.059–1089.488) 1079.547 (908.632–1396.909) 0.18^§^	1.00^‡^ 1.00^‡^
MDA (nmol/ml) Baseline End point p-value of within group	7.709 (6.5–8.808) 7.337 (6.378–9.145) 1.00^§^	7.465 (5.477–8.221) 8.419 (6.453–9.477) 0.62^§^	0.94^‡^ 1.00^‡^
IL-1 (ng/l) Baseline End point p-value of within group	35.6 (14.475–39.425) 42.45 (24.725–50.525) 0.064^§^	34.2 (29.1–48.75) 34.5 (19.7–40.9) 0.20^§^	1.00^‡^ 0.26^‡^
IL-6 (ng/l) Baseline End point p-value of within group	64.167 (53.583–83.708) 68 (53.333–80.292) 1.00^§^	70.5 (56.667–84.167) 79.667 (65.667–87.584) 1.00^§^	1.00^‡^ 0.68^‡^
hs-CRP (mg/ml) Baseline End point p-value of within group	10.057 (9.779–10.331) 9.721 (6.102–10.191) 0.032^†^,^§^	9.903 (8.481–10.482) 9.509 (7.712–9.856) 1.00^§^	1.00^‡^ 1.00^‡^
TNF-α (mg/ml) Baseline End point p-value of within group	99.125 (55.375–136.063) 102.5 (88.688–139.563) 0.60^§^	101.25 (82–136.875) 128.75 (95.75–162.625) 0.056^§^	1.00^‡^ 0.61^‡^

Data are expressed as median (interquartile range).

† A p-value 0.05 denotes significant difference.

‡ Mann–Whitney *U* test.

§ Wilcoxon signed ranks test.

hs-CRP: High-sensitivity C-reactive protein; IL-1: Interleukin-1; IL-6: Interleukin-6; MDA: Malondialdehyde; sVCAM-1: Soluble vascular cell adhesion molecule-1; TNF-α: Tumor necrosis factor alpha.

### Incidence of major adverse cardiac events

Comparison of the incidence of MACEs between the two groups concluded no difference regarding the total MACEs incidence six (27.3%) versus nine (42.9%), p-value = 0.35). Most MACEs were recurrent anginal pain and only one patient in the placebo group suffered a second MI, as shown in Table 3.

**Table 3.** Comparison of efficacy and safety between the pentoxifylline and the placebo groups.

Parameter	Drug group (n = 22)	Placebo group (n = 21)	p-value
Total MACEs Anginal pain Myocardial Infarction	6 (27.3%) 6 (27.3%) 0 (0%)	9 (42.9%) 8 (38.1%) 1 (4.8%)	0.35^†^ 0.45^†^ 0.49^‡^
Side effects Nose bleeding Headache Nausea	0 (0%) 6 (27.3%) 3 (13.6%)	1 (4.8%) 3 (14.3%) 1 (4.8%)	0.49^‡^ 0.25^‡^ 0.32^‡^
Adherence	90.80% ± 6.1	87.81% ± 5.42	0.10^§^

Data are expressed as number (%) or mean ± SD.

† Pearson Chi-square test.

‡ Fisher's Exact test.

§ unpaired t-test.

MACE: Major adverse cardiac event; PTX: Pentoxifylline.

### Assessment of safety and tolerability of pentoxifylline

The use of PTX in the trial was well tolerated. No significant side effects were reported with only one patient experiencing nose bleeding in the placebo group. A few patients reported headache or nausea, the incidence of which is shown in Table 3.

Adherence of the patients to either PTX or placebo was compared as shown in Table 3.

## Discussion

To address the need for new treatment strategies to improve the outcomes of STEMI patients, we attempted to investigate PTX treatment for 2 months in 43 STEMI patients who had undergone PCI. The effect of PTX on ED, oxidative stress, inflammation as well as MACEs development was studied.

Our study demonstrated that PTX administration for 2 months in STEMI patients elicited no significant difference on neither the levels of sVCAM-1 as a marker for ED, MDA as a marker for oxidative stress nor the inflammatory markers IL-1, IL-6, hs-CRP and TNF-α among the study groups. However, the within-group comparison revealed a statistically significant reduction in the level of hs-CRP in the PTX group only. PTX also showed a neutral effect on MACEs development.

Soluble vascular cell adhesion molecule-1 (sVCAM-1) and soluble intercellular adhesion molecule-1 (sICAM-1) are both elevated in STEMI patients [31]. However, we selected sVCAM-1 as the biomarker for the assessment of endothelial function since it is reported to be a better predictive marker of cardiovascular events in patients with ACS [32]. It was also observed that sVCAM-1 rises even before obstruction occurs and thus it could improve the predictive accuracy of MACEs risk in STEMI patients [31,32].

The starting baseline levels of sVCAM-1 in the present study were significantly and positively correlated with the age of the participants which is in line with previous studies [33,34]. This may be explained by the age-related buildup of reactive oxygen and nitrogen species followed by a decline in total thiol content, that would lead to a net increase in oxidative stress. As a result, vascular endothelial cells activation occurs with high quantities of adhesion molecules secreted, and the likelihood of developing vascular problems in the elderly population increases [35].

Our finding that PTX had no effect on ED is in line with Bilsborough *et al.* They administered PTX with a dose of 400 mg/day three times daily for 8 weeks in patients with type II diabetes where PTX did not result in any significant improvement in ED as evaluated by flow-mediated dilation (FMD) despite the reduction observed in the TNF-α level. They suggested that TNF-α could be important in contributing to ED in more severe or more obese, diabetic cases [36]. Moreover, the two studies by Gupta *et al.* were in concordance with the current study. In HIV patients not treated with antiretroviral therapy, PTX administration for 8 weeks did not significantly improve FMD, sVCAM-1, IL-6, hs-CRP or TNF receptor-1 [37]. Also, in HIV patients receiving antiretroviral treatment and PTX for 48 weeks, PTX had no effect on FMD, sVCAM-1 or hs-CRP with a slower decrease in the level of IL-6 observed in the PTX group compared with the placebo group [38]. The researchers proposed that more severe cases may derive more benefit from PTX treatment. Despite the similarity between the findings of the current study and the aforementioned ones, we must acknowledge the variability between our patients and theirs that may hinder an accurate comparison. What is important to consider is the small number of patients recruited in the three previously summarized studies as well as the relatively small sample size of the current trial. We also did not examine our patients' FMD and we acknowledge that a bigger sample size may have increased the ability to detect differences and improved analyses of the biomarker alterations when examining them in certain subgroups.

On the contrary, in a study that used PTX for 2 months in patients with angiographically proven atherosclerosis, the reduction in the level of sVCAM-1 was significantly higher in the PTX than the placebo group [30]. We hypothesize that the disparity between our findings and former trial's results may be due to the difference in the kinetics of sVCAM-1 between STEMI patients and their study participants with less severe CAD. It was observed that the levels of sVCAM-1 in STEMI patients continued to increase until 1 month post-MI [39]. Furthermore, in patients with unstable angina or non-Q-wave infarction, the levels of these soluble adhesion molecules persisted for weeks to months after the recovery of the clinical symptoms. Only 6 months later did their levels approach control levels. This is in line with the hypothesis that maximum inflammation is reached sometime after the event and not at admission [40]. Although we proposed that PTX would abrogate or at least attenuate the prolonged increase in ED and inflammation, however the obtained result draws the conclusion that a longer duration of treatment with PTX is required to illustrate a response.

Regarding the effect of PTX on the inflammatory markers, PTX did not show a significantly different effect on the levels of IL-1, IL-6, TNF-α or hs-CRP between the two groups. Only the level of hs-CRP was significantly decreased compared with its baseline value in the PTX group. In contradiction to our findings, Fernandes *et al.* reported that the administration of PTX in NSTEMI patients for 6 months in the FDA-approved dose resulted in a significant decrease in the level of CRP and TNF-α. Nevertheless, they did not obtain a significant difference regarding IL-6 which is the same in the current study [21]. Later, the results of a meta-analysis on PTX concluded that it exerted a significant reduction effect on the levels of TNF-α and CRP in disorders such as CAD where the reduction in TNF-α was correlated with the treatment duration but not the PTX dose. That same meta-analysis found that PTX had no significant effect on the level of IL-6 across the investigated studies which is in accordance with our study results [41]. A more recent study examined the administration of PTX in NSTEMI patients and observed that PTX attenuated the rise of hs-CRP but without a significant difference in its level compared with the placebo group [22]. The inconsistent outcomes from different trials as well as the small number of studies performed on ACS patients, especially STEMI, endorse our previous conclusion that larger studies with an extended duration of treatment with PTX are required to obtain a firm conclusion.

The current study did not observe an improvement in MDA plasma levels by PTX treatment. However, when plasma MDA was assessed in hypertensive diabetic patients using PTX in a dose of 400 mg daily for 4 weeks, a significant decrease in its level was observed [42]. To our knowledge, no studies attempted to evaluate the effect of PTX on MDA levels in CAD or ACS patients. Several animal studies also revealed inconsistent outcomes regarding the effect of PTX on MDA levels [^43-46^]. On the other hand, a study in hepatic ischemia reperfusion injury raised questions about PTX's antioxidant benefits when it revealed its neutral effects on MDA levels [47]. However, all performed studies are relatively small and lack sufficient power to detect most of the outcomes.

Pentoxifylline dose at 1200 mg/day is the maximum well-tolerated and safe dose with higher doses causing more pronounced gastrointestinal side effects [48]. In the current study, PTX in STEMI patients did not have any pronounced side effects and was thus well-tolerated. The occurrence of nausea as a gastrointestinal side effect and headache was comparable between the two study groups.

Adherence of the current study patients to both PTX and placebo tablets exceeded the a cut-off of ≥80%, implying adherence as per the results of the study by Bansilal *et al.* which concluded that an adherence level of 80% or more to cardiac medications immediately following an MI was associated with 27% lower risk of MACEs than nonadherent patients [49].

At the end of the 2 months follow-up period, there were no statistically significant differences regarding the occurrence of MACEs between the two groups. The same result was observed in a trial that utilized PTX in STEMI patients albeit undergoing thrombolytic therapy as it found no significant difference in the 30-Day MACEs occurrence between PTX and placebo group [50]. The more recent study by Brie *et al.* that used PTX on NSTEMI patients in the same dose did not show a significant reduction in the development of MACEs at 1 year [22]. Whether the same PTX neutral impact on MACEs in STEMI patients will be obtained after long-term follow-up needs further studying in larger outcome-based clinical trials.

The results of this study need to be interpreted with caution in light of some limitations. Due to logistical constraints, our trial was single-blinded and single-centered. As the investigator was aware of patient allocation, potential reporting and observer bias could have occurred and hence it is recommended that future double-blinded trials be conducted. Recruiting patients from a single hospital may affect the generalizability of the study results. Future multi-centered trials are recommended to confirm the results in larger populations. The short duration of the study and the lack of long-term follow-up post-study may have truncated the outcomes at that time point and further effect could be potentially illustrated in future longer trials. Hence, we report our results only pertaining to the duration of the study of 2 months and propose the need for a longer study duration with the same population. Even though our trial was fully powered to detect the primary outcome based on the sample size calculated from a previous study, the relatively small sample size warrants a future larger trial to be carried out.

The choice of antiplatelets used after the event in our patients varied between ticagrelor or clopidogrel depending on the availability in the hospital pharmacy which could be viewed as a limitation as there was a statistically significant difference in the distribution of both drugs between the PTX and the placebo group. However, the two antiplatelets haven't been proven to be different regarding their anti-inflammatory effect [51]. Also, in the Hunting for the Off-Target Properties of Ticagrelor on Endothelial Function in Humans (HI-TECH) trial on stable patients following ACS, ticagrelor had no significantly different effect on markers of ED including FMD and sVCAM-1 [52]. Even with considering the possibility of a favorable profile with ticagrelor, the study outcomes were not tipped in favor of PTX despite the presence of slightly more patients who were administered ticagrelor in the PTX group versus the placebo group. Despite such a limitation being incidental in our study, similar trials performed ahead need to avoid such unbalanced distribution to avoid any confounding effect.

## Conclusion

The study results showed that PTX administration for 2 months had no significant effect on ED as measured by sVCAM-1, oxidative stress as measured by MDA or inflammatory markers as measured by IL-1, IL-6, TNF-α and hs-CRP without affecting MACEs development. However, PTX administration 1200 mg/day in STEMI patients was safe and well tolerated during the study duration.

Given the neutral effects of PTX on the investigated parameters, it seems that there is no sufficient evidence for firm recommendations to be drawn regarding the future incorporation of PTX in the clinical management of STEMI patients yet. However, the significantly improved inflammatory marker hs-CRP in STEMI patients who have received PTX coupled with the observed tolerable use of PTX in our study present an opportunity for further clinical trials to be performed using PTX in STEMI patients. It is recommended that a larger study with a longer duration of treatment with PTX and longer period of follow-up be conducted to elucidate its firm effects on the biomarkers as well as the MACEs. Direct testing for vasomotor function and thus endothelial function in the infarct-related artery could also reveal more accurately any effects of PTX on ED.

## Data sharing statement

The authors certify that this manuscript reports original clinical trial data. Deidentified, individual data that underlie the results reported in this article (text, tables, figures and appendices) will be available for anyone who wants access to them upon request.
